# The effect of the administration form of antibiotic therapy on the gut microbiome in patients with infected diabetic foot ulcers - DFIATIM trial

**DOI:** 10.1186/s12866-025-04041-0

**Published:** 2025-05-28

**Authors:** Chahrazed Mekadim, Jakub Mrazek, Kateřina Olša Fliegerová, Hana Sechovcová, Tiziana Maria Mahayri, Radka Jarošíková, Jitka Husáková, Veronika Wosková, Petr Tůma, Jan Polák, Dominika Sojáková, Andrea Němcová, Michal Dubský, Vladimíra Fejfarová

**Affiliations:** 1 Laboratory of Anaerobic Microbiology, Institute of Animal Physiology and Genetics, Czech Academy of Sciences, v.v.i, Videnska 1083, Prague, 142 00 Czech Republic; 2 Diabetes Centre, Institute for Clinical and Experimental Medicine, Prague, Czech Republic; 3 Department of Internal Medicine, Second Faculty of Medicine, Charles University, Prague, Czech Republic; 4 Department of Hygiene, Third Faculty of Medicine, Charles University, Prague, Czech Republic; 5 Department of Pathophysiology, Third Faculty of Medicine, Charles University, Prague, Czech Republic

**Keywords:** Diabetic foot infection, Diabetic foot ulcers, Diabetes, Gut microbiota, Antibiotics, Beta-lactam, Bolus, Continuous

## Abstract

**Background:**

Diabetic foot infections (DFIs) contribute to the global disability burden. Beta-lactams are the most commonly used antibiotics for treating DFIs. However, the use of antibiotics may lead to disruption of the healthy balance of the gut microbiota, causing dysbiosis.

**Methods:**

Patients with infected diabetic foot ulcers (iDFUs) were treated with two kinds of beta-lactams (amoxicillin/clavulanic acid or ceftazidime) according to microbial sensitivity of causative agents via bolus or continuous administration modes. Changes in the gut microbiome of patients were analyzed. Diabetic patients without iDFUs were used as a control group. 16 S ribosomal RNA gene amplicon sequencing was performed on stool samples collected from participants.

**Results:**

Alpha diversity and beta diversity of gut microbiota of treated patients did not show significant differences between bolus and continuous modes. However, significant differences were observed between gut microbiota diversity of treated patients and control group. PCoA plots showed individualized responses of the patient’s gut microbiota to antibiotics at different times using both administration forms associated with the pre-treatment state of microbiota composition. *Enterococcus*, *Sellimonas*, and *Lachnoclostridium* were the common bacterial markers differentially abundant in the gut microbiota of antibiotic-treated patients with iDFUs while *Roseburia*, *Dorea*, and *Monoglobus* were mainly abundant in the gut microbiota of patients without iDFUs. Predicted pathways like “Transporters”, “ABC transporters” and “Phosphotranspherase system (PTS)” were upregulated in the gut microbiome of patients treated with bolus regime which may lead to increased intestinal barrier permeability.

**Conclusion:**

The present study reported alterations in gut microbiota composition and functionality and provided the bacterial markers as well as potential metabolic signatures associated with each administration mode in patients with iDFUs, which may be used as a reference set for future studies of the effect of antibiotics administration on the gut microbiome of patients with iDFUs. This study shed light on the importance of understanding the effect of antibiotic administration form on gut microbiome in patients with iDFUs.

**Trial registration:**

The DFIATIM Clinical Trial (Full title: “Rationalisation of ATB therapy in diabetic foot infection and its impact on the intestinal microbiota”) is submitted to the European Union Clinical Trials Database under the EudraCT Number: 2019-001997-27. The date of registration is July 17th, 2020.

**Supplementary Information:**

The online version contains supplementary material available at 10.1186/s12866-025-04041-0.

## Introduction

Diabetic foot infections (DFIs) are the most frequent complications in patients with diabetes mellitus (DM). The majority of DFIs occur due to inadequately controlled diabetes, peripheral neuropathy associated with loss of protective sensation and reduced peripheral arterial flow [1–4]. Injure in the skin enables the entry of microorganisms which lead to colonization and damage of host tissue which can manifest rapidly as a clinical infection [5]. Based on IWGDF/IDSA criteria DFIs could be categorized into mild, moderate, and severe infections [6]. Untreated iDFUs may cause progression of infection leading to more severe complications requiring lower limb amputations to prevent the spreading of infection that could be life-threatening. Hospital admissions of patients with DFIs are correlated with a high death rate and high financial costs of treatment, which drive a considerable burden on health care [7]. Data from the Institute of Health Information and Statistics of the Czech Republic (covering the year ending Dec. 31, 2021) show a continuing trend of increased incidence of diabetes mellitus, which affected over 8% of the population [8]. According to the Institute of Health Information and Statistics of the Czech Republic, there were 1,065,178 patients with diabetes in 2021. At the same time, 60,433 subjects (5.7%) suffered from diabetic foot in the Czech Republic. Nearly 0.4% (4739) of all patients with diabetes annually underwent lower limb amputations however, from those nearly 45% were absolved in 2021 major amputation [8]. Therefore, early detection of the possible pathogenesis agent of DFIs and prompt treatment are important for better management of DFIs in order to improve outcomes via a multidisciplinary approach.

Antibiotics are, usually, necessary for treating foot infections in individuals with diabetes to reverse and resolve DFIs and thereafter promote wound healing and prevent complications. The antibiotic needs to be selected according to detected pathogens and severity of infection, previous antibiotic treatment and DFIs caused by antibiotic-resistant organisms, and the patient’s clinical response to the antibiotic [9, 10]. Beta-lactam is a large group of antibacterials frequently used for treating DFIs including two principal classes: Penicillins and Cephalosporines [11, 12]. DFIs antibiotic therapy is mostly empirical based on the type of microbial agent, the severity of DFIs, expected treatment duration and the mode of administration. The moderaty and severity of DFIs, especially of iDFUs, should be treated by parenteral antibiotics [6]. Two primary types of antibiotic administration routinely applied are mostly bolus and less frequently continuous modes. Bolus administration refers to the administration of antibiotics in a single high dose of the antibiotic multiple times a day in order to maintain therapeutic levels. This approach causes the highest concentrations of antibiotics in the blood at the start, followed by a decline as the drug is eliminated from the body. Unlike bolus (or intermittent) administration, continuous (or extended) administration requires infusing the antibiotic gradually to ensure a sustained level of antibiotic in the bloodstream over a longer period. The form of administering antibiotics (bolus or continuous) depends on several factors including the pharmacokinetic properties of the antibiotic, the severity of the infection, the comorbidities of the patient and the type of healthcare setting. Normally, guidelines recommend predominantly bolus antibiotics, especially in time-dependent antibiotics in which the beta-lactams class belong. Even though bolus administration brings to the reach effective therapy rapidly, continuous administration could potentially offer more sustained and consistent levels of the antibiotic in the bloodstream, which may probably reduce the risk of toxicity and optimize treatment efficacy and outcomes [13]. Beta-lactam antibiotics provide an instance of drug class where continuous administration is recommended as another strategy to the conventional intermittent bolus dosing in order to optimize the pharmacokinetic/pharmacodynamic (PK/PD) properties. Prolonged (or extended) infusions of beta-lactam antibiotics are preferable for bacteria with high minimum inhibitory concentration (MIC) to achieve sufficient time above the MIC [14–16].

Though the use of antibiotics is crucial for the treatment of DFIs, it may lead to various effects on the gut microbiome which is the community of microorganisms located in the gut that play an essential role in host health [17]. Findings have revealed that antibiotics used for targeting pathogenic bacteria can consequently disrupt the healthy balance of the gut microbiota causing dysbiosis, through reducing microbial diversity, altering the microbiota composition, and promoting the proliferation of antibiotic-resistant strains [17–20]. The alterations in the gut microbiome of patients with DFIs, especially those with iDFUs, as they frequently need to apply prolonged or multiple administrations of antibiotics, may have systemic consequences to their health and metabolic function [18, 21–25]. Furthermore, gut microbiota dysbiosis has been linked to the pathogenesis of T2DM and its complications [26], suggesting that modulation of the gut microbiome could be a promising therapeutic strategy in the treatment of DM [27–32]. Emerging evidence suggests interventions targeting the gut microbiota that restore microbial balance such as probiotics, prebiotics, synbiotics and fecal microbiota transplantation (FMT) may exert beneficial effects on the management of diabetes and associated complications [33–41] including wounds healing in patients with DFIs [42, 43]. Recently, numerous research findings have demonstrated that not only changes in the commensal skin microbiome may contribute to the development of cutaneous wounds but also alterations in the gut microbiome are associated with various skin diseases [44]. Interestingly, it was shown that probiotic supplementation for 12 weeks among diabetic foot patients reduced diabetic foot ulcer size [45]. Thus, the association between dysbiosis of the gut microbiota and DM allow us to propose a potential bidirectional relationship between antibiotic use, gut dysbiosis, and DFIs via the gut-skin axis. The precise mechanism underlying the gut microbiome-skin interactions is still unrevealed [46].

Specific data on the effect of antibiotic administration on the gut microbiota of patients with DFIs are very rare. Therefore, the aim of the present study is to assess the effects of administration of two types of beta-lactam antibiotics (amoxicillin/clavulanic acid and ceftazidime) via bolus and continuous mode on the gut microbiome in patients with iDFUs.

## Materials and methods

### Patients, treatment and specimen collection

Entirely, we enrolled 60 patients with iDFUs (mean age 65 ± 9 years, HbA1c 64 ± 19 mmol/mol, BMI 32.7 ± 5.1 kg.m^− 2^, serum creatine 96 ± 36 umol/L and CRP 23 ± 40 mmol/L) into the DFIATIM study between 9/2020 and 9/2024. They were treated for diabetic foot infection with signs of infection at least of moderate stage described in international guidelines for diabetic foot management [6]. Patients had to have at least erythema extending ≥ 2 cm from the wound margin and/or tissue deeper than skin and subcutaneous tissues (wound depth touching tendon, muscle, joint, and/or bone) and/or osteomyelitis and/or systematic signs of infection. These study subjects were divided into two groups based on the type of used antibiotics: 30 patients treated with amoxicillin/clavulanic acid (AMC) (1.2 g every 8 h for bolus or continuously 3.6 g applied during 24 h) and 30 patients treated with ceftazidime (CTZ) (2.0 g every 8 h for bolus or continuously 6 g applied during 24 h) according to causative agents found in iDFUs. Patients in both study groups were subdivided into two treatment arms based on randomization those treated by intravenous bolus or continuous antibiotic therapy using the same dosage of antibiotics per day.

**Fig. 1:**
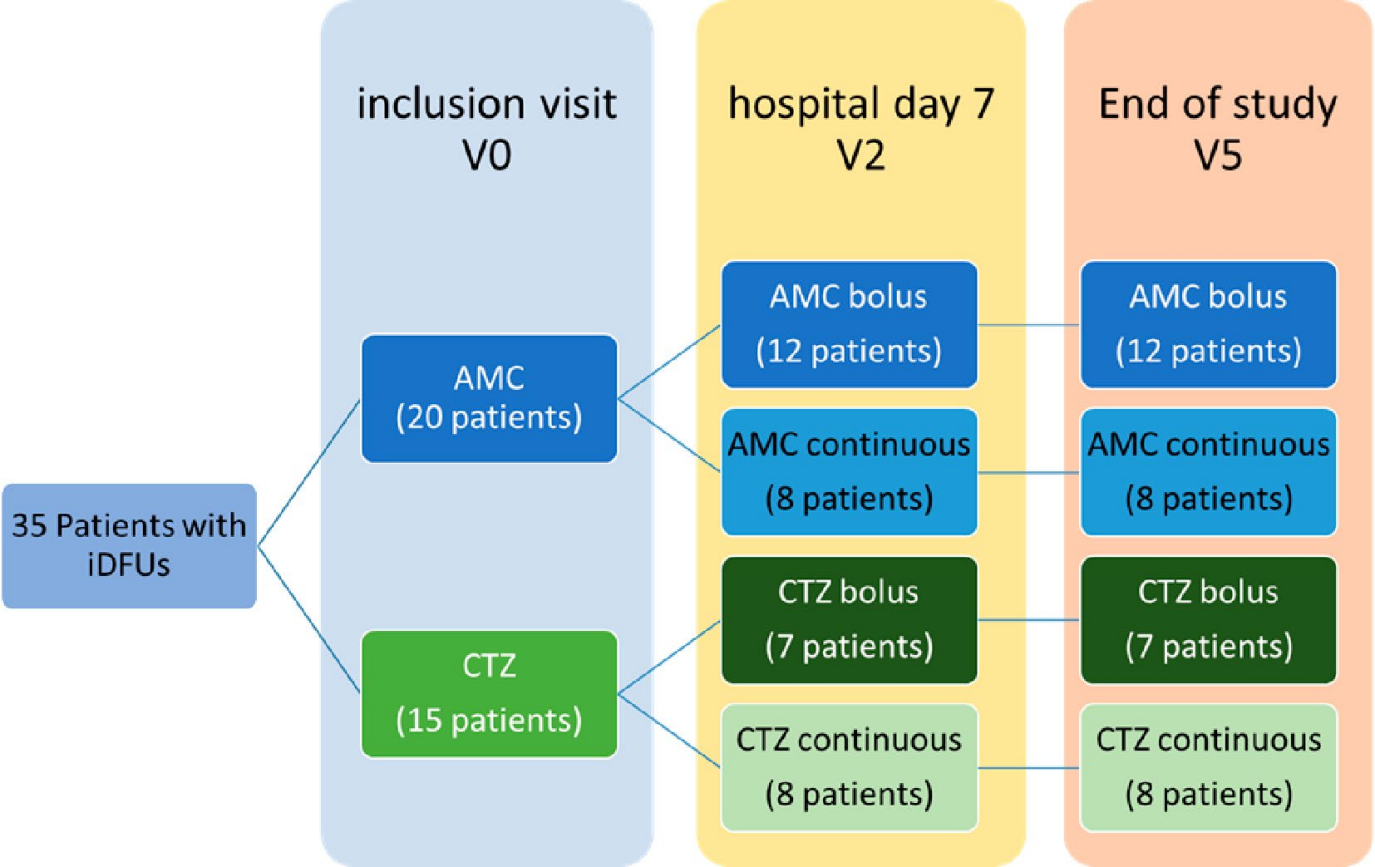
Experimental scheme of the study workflow showing patients with iDFUs treated with amoxicillin/clavulanic acid (AMC) and ceftazidime (CTZ) using bolus and continuous administration modes and different times of collection of stool: V0 (hospital day 0– inclusion visit), V2 (hospital day 7) and V5 (End of study– 8weeks after hospital discharge)

All study subjects were admitted and treated for at least seven days by parenteral antibiotics. During inclusion day (hospital day 0– inclusion visit– V0) and at the end of hospitalization (hospital day 7– V2) planned examinations based on the study schedule including labs, serum and collection of stool samples. After the hospital discharge, patients with iDFUs were managed comprehensively and followed for the next 8 weeks, when the DFIATIM trial was finished (V5). Treatment was conducted according to the DFIATIM trial scheme prepared by Fejfarová et al. [47]. The experimental scheme of the study workflow is described in Fig. 1. The DFIATIM trial has been approved by the ethics committees of the Institute for Clinical and Experimental Medicine and Thomayer University Hospital (The Czech Republic). This study adheres to CONSORT guidelines (see the CONSORT checklist).

Only 35 patients provided stool samples at all three times of collection. Patients with missed stool samples were excluded from the present study. In total, 105 stool samples were collected from 35 patients. 36 samples from patients treated with AMC bolus (12 patients), 24 samples from patients treated with AMC continuous (8 patients), 21 samples from patients treated with CTZ bolus (7 patients) and 24 samples from patients treated with CTZ continuous (8 patients). Stool samples also were collected from 17 diabetic patients to be used as controls. These patients fulfilled similar inclusion criteria as those patients included in DFIATIM trial [47], however, they did not suffer from DF and were at least 3 months of antibiotics naïve before inclusion into our study. (Details of all patients are listed in Table S1).

### DNA isolation and sequencing of 16 S rDNA amplificons

DNA was extracted from stool samples using the QIAamp PowerFecal Pro DNA Kit (QIAGEN, Germany) according to the manufacturer’s protocol. The eluted DNA was used to prepare amplicons of V4-V5 region of the 16 S rRNA gene using the following PCR conditions: denaturation for 5 min at 95 °C, followed by 34 cycles of 30 s at 95 °C, 30 s at 57 °C and 30 s at 72 °C, ending with a final elongation for 5 min at 72 °C. The quality of the PCR amplicons was checked by electrophoresis in 1.5% agarose gel (30 min at 100 V), then the amplicons were purified using a Monarch PCR & DNA Cleanup Kit (New England Biolabs, USA) according to the manufacturer’s protocol and the concentration of the purified amplicons was determined using a Nanodrop OneC Microvolume UV-Vis Spectrophotometer (Thermo Scientific, USA).

Libraries were prepared from purified amplicons of the V4-V5 region of the 16Sr RNA gene (300 bp) using the NEBNext^®^Fast DNA Library Prep Kit (New England Biolabs, USA) according to Milani et al. 2013 [48]. The sequencing was then performed on the Ion Torrent platform (Termo Fisher Scientifc, USA) as it was described previously by Mekadim et al. [49].

### Microbiome analysis and statistical analysis

Bacterial 16 S rRNA gene sequences were obtained in FASTQ format and analyzed using QIIME 2 version 2022.2 [50]. Briefly, quality filtering, chimera check, and trimming of sequences were performed by the DADA2 [51]. Then, the obtained amplicon sequence variants (ASVs) were taxonomically classified using VSEARCH based on SILVA database (version 138) with a 99% threshold of similarity [52]. The rarefaction was performed based on the sequence depth to normalize data. The α-diversity was determined using pielou evenness, Shannon, Chao1, and Simpson’s diversity indices based on the Kruskal–Wallis test. The β-diversity was indicated using Principal Coordinate Analysis (PCoA) based on Bray Curtis distance. The box plots for α-diversity indices and the 2-dimensional PCoA plots were generated in R-Studio (http://www.rstudio.com/) using ggplot2 (https://ggplot2.tidyverse.org) packages. Ellipses mark 95% of confidence for each group and *p* ≤ 0.05 was considered statistically significant. Permutational multivariate analysis of variance (Adonis) and Bray Curtis distance matrix were used to evaluate the dissimilarity among groups with permutation set at 999. The linear discriminant analysis with effect size (LEfSe) algorithm was performed based on Kruskal–Wallis test and the pairwise Wilcoxon test to identify genera with significant differential relative abundances between groups with α value of 0.05 and threshold value at 2.0. PICRUSt2 [53] was applied for pathway functional prediction using KEGG database at levels 1, 2 and 3. STAMP program [54] was utilized for statistical analysis by using Non-corrected Welch’s t-test type two-sided, with a confidence interval (CI) of 0.95 with *p* < 0.05 was considered to be statistically significant.

## Results

### Gut microbiome analysis

The bacterial profile analysis was performed on a total of 122 stool samples from control participants (17 samples) and patients who received two different types of antibiotic therapy (AMC and CTZ) in two administration forms (continuous and bolus) (Table S1). A total of 8,291,294 sequence reads were obtained from samples of all patients. The mean sequence length was 267 bp. Sequences were deposited into the SRA database of NCBI under accession number PRJNA1071358.

#### Effects of antibiotic administration on the gut bacterial diversity of patients with iDFUs

**Fig. 2:**
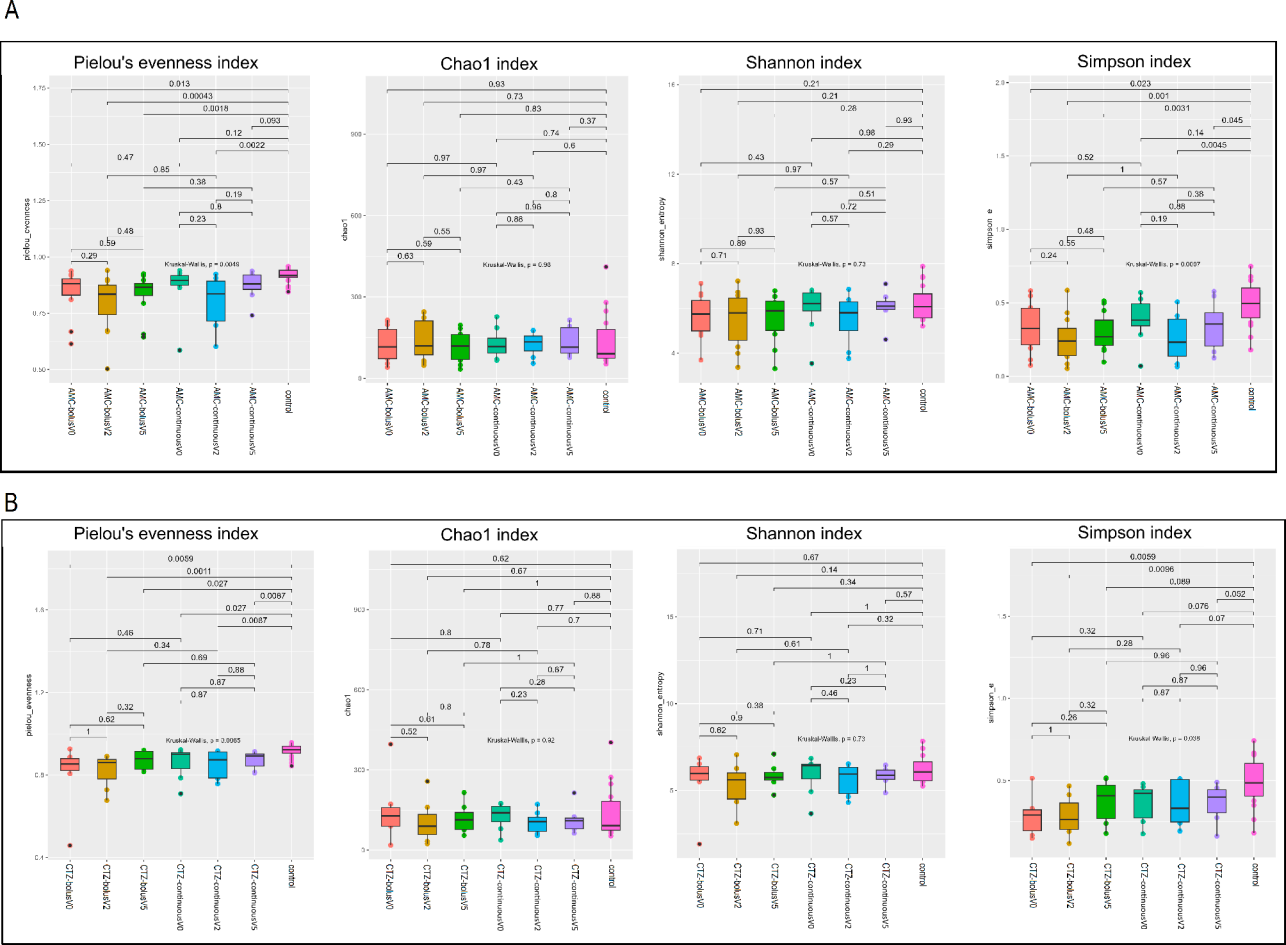
Boxplots illustrating pielou_evenness, chao1, shannon and simpson diversity indices in bacterial community of fecal microbiome of control participants and patients with iDFUs treated with (**A**) amoxicillin/clavulanic acid (AMC), (**B**) ceftazidime (CTZ) using bolus and continuous administration modes at different times of collection V0 (before hospitalization), V2 (one week after hospital admission) and V5 (two months after hospital discharge), p-value ≤ 0.05 was considered statistically significant based on the Kruskal-Wallis test

Alpha diversity analysis using pielou evenness, chao1, shannon and simpson indices showed no significant difference in the richness and the evenness of gut microbiome diversity between different times of sample collection (V0, V2 and V5) in both antibiotics treatments (AMC and CTZ) and administration forms (bolus and continuous) (Fig. 2A, B). Significant differences were observed between the gut microbiome of the control group and some groups of treated patients using pielou evenness and simpson indices but not chao1and shannon indices (Fig. 2A, B).

**Fig. 3:**
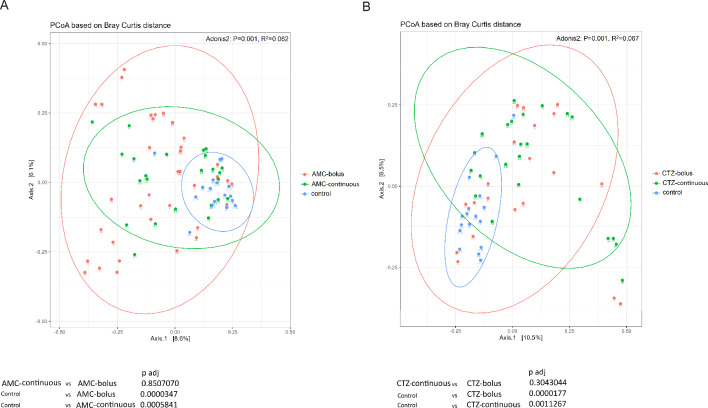
Principal Coordinate Analysis (PCoA) plots based on Bray Curtis distance of fecal microbiome from control participants and patients with iDFUs treated with: (**A**) amoxicillin/clavulanic acid (AMC), (**B**) ceftazidime (CTZ), Label numbers correspond to the patient’s ID. Dissimilarity analysis between the two groups was performed using Adonis with permutation 999. The confidence ellipses were traced in the 95% confidence. p-value ≤ 0.05 was considered statistically significant

Beta diversity analyses using PCoA based on Bray Curtis distance revealed significant differences between the control group and treated patients group using two administration forms: bolus and continuous, in both antibiotic treatments AMC and CTZ (Fig. 3A, B). However, no significant difference was observed between different times of sample collection (V0, V2 and V5) in both antibiotics treatments and administration forms (Fig. S1). Inter-individual variation in gut microbiota diversity is demonstrated by shorter distances between samples from the same patient compared to different patients. The difference in beta diversity was inter-patients and not intra-individuals at different times (V0, V2 and V5) (Fig. S1).

#### Effects of antibiotic administration on the gut bacterial composition of patients with iDFUs

**Fig. 4:**
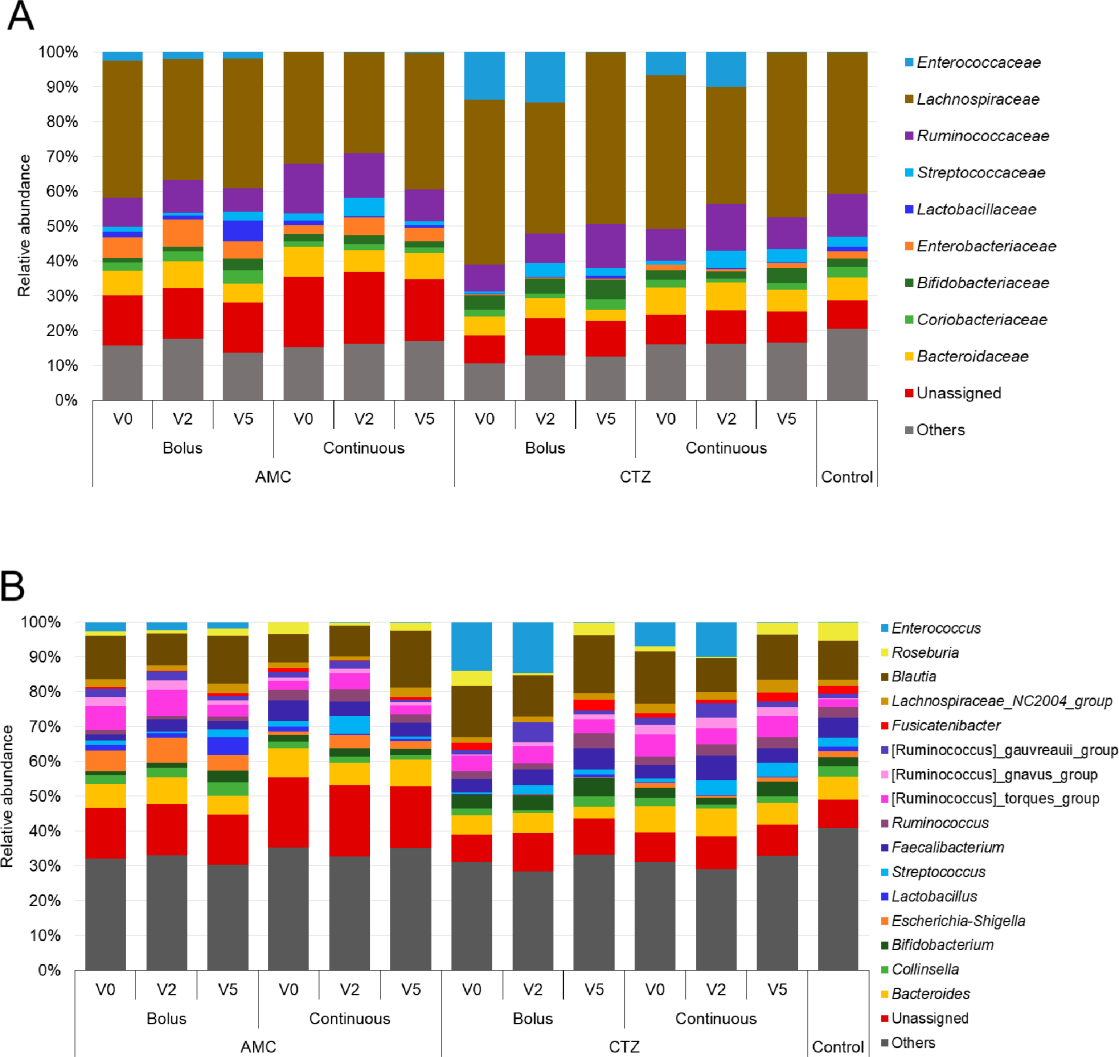
Relative abundance of bacterial populations at (**A**) family and (**B**) genus levels of fecal microbiomes of control participants and patients with iDFUs treated with amoxicillin/clavulanic acid (AMC) and ceftazidime (CTZ) using bolus and continuous administration modes at different times of collection V0 (before hospitalization), V2 (one week after hospital admission) and V5 (two months after hospital discharge)

The relative abundance of gut bacterial composition was assessed at phylum, family and genus levels to investigate the effects of antibiotic administration on the gut bacterial composition of patients with iDFUs (Figs. 4 and S2, Tables S2 and S3).

At the phylum level (Fig. S2), four bacterial phyla were identified: *Bacillota* (formerly named *Firmicutes*), *Bacteroidota* (formerly named *Bacteroidetes*), *Pseudomonadota* (formerly named *Proteobacteria*) and *Actinobacteriota* (formerly named *Actinobact*eria). Figure 4 shows the relative abundance of bacteria, at family (A) and genus (B) levels, in the stool samples of control participants and patients according to the time of collection (V0, V2 and V5) in each administration form (bolus and continuous) using two antibiotics (AMC and CTZ). *Bacillota* was found to be the dominant phylum in all samples. The main class of *Bacillota* was *Clostridia*, which was represented at the family level by *Lachnospiraceae* and *Ruminococcaceae*. The class *Bacilli* was represented by the families *Enterococcaceae*, *Streptococcaceae* and *Lactobacillaceae* (Fig. 4A). The phylum *Bacteroidota* was mainly represented by the family *Bacteroidaceae*. The phylum *Actinobacteriota* was mainly represented by the families *Coriobacteriaceae* and *Bifidobacteriaceae*. *Pseudomonadota* phylum was the lowest abundant bacterial phylum in patients treated with CTZ. *Enterobacteriaceae* was the main family of *Pseudomonadota* phylum. *Enterobacteriaceae* were higher in patients treated with AMC while *Enterococacceae* were higher in patients treated with CTZ (Fig. 4A).

At the genus level (Fig. 4B), *Bacteroides*,* Blautia*,* Faecalibacterium*,* Ruminococcus*,* Roseburia*,* Streptococcus*, and *Collinsella* were the most abundant bacterial genera in the fecal microbiome of all studied patients. *Lactobacillus* was higher in fecal microbiome of patients treated with AMC using bolus administration form. The abundance of *Bifidobacterium* was higher in fecal microbiome of all study individuals treated with CTZ. *Escherichia*-*Shigella* were significantly higher in fecal microbiome of patients treated with AMC compared to the fecal microbiome of patients treated with CTZ. Interestingly, the abundance of *Escherichia*-*Shigella* increased after one week of treatment with AMC (V2). *Escherichia*-*Shigella* were significantly higher in fecal microbiome of patients treated with CTZ using continuous form than in the fecal microbiome of patients treated with CTZ using bolus form (*P* = 0.026) (Table S3). *Enterococcus* was significantly higher in fecal microbiome of patients treated with AMC using bolus administration form than in fecal microbiome of patients treated with AMC using continuous administration form (*P* = 0.007) and the control group (*P* = 0.006) (Table S3). *Enterococcus* was reduced dramatically at V5, approximately 10 weeks after CTZ treatment, using both administration forms. This decrease in *Enterococcus* abundance was associated with a significant increase in the relative abundance of *Blautia*, *Roseburia*, *Ruminococcus Fusicatenibacter* and *Feacalibacterium* (Fig. 4B).

**Fig. 5:**
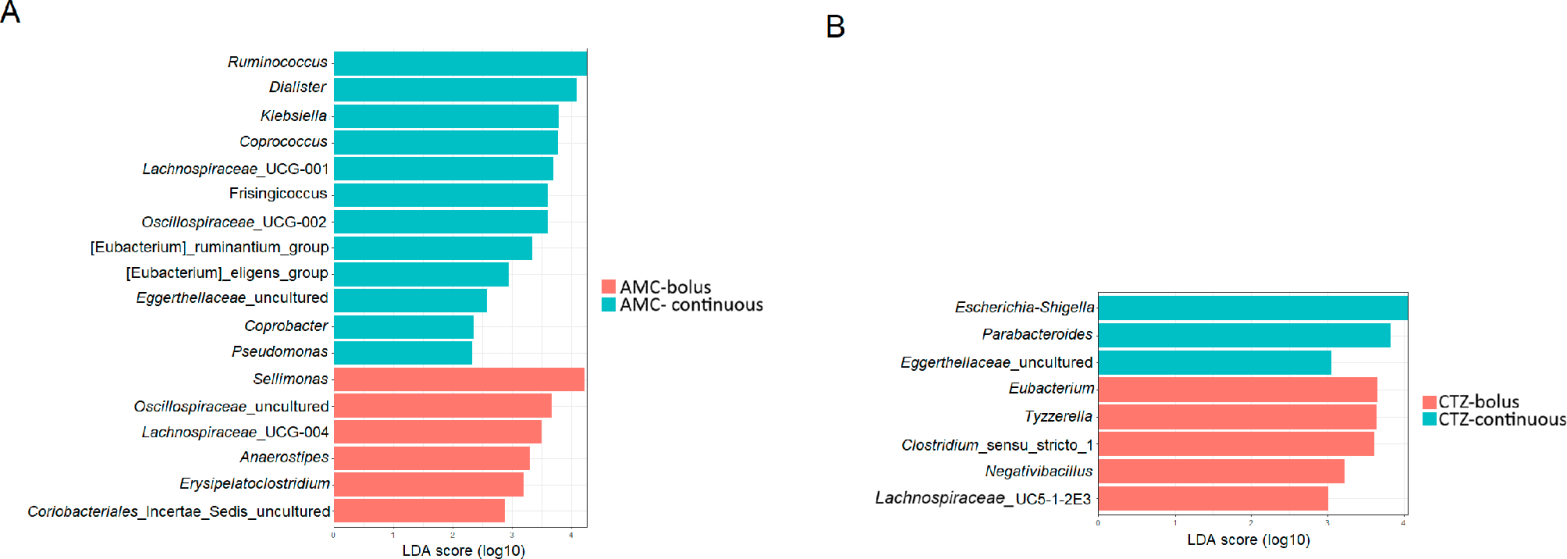
Linear discriminant analysis effect size (LEfSe) of taxa at genus level in fecal microbiomes from patients with iDFUs treated with (**A**) amoxicillin/clavulanic acid (AMC), (**B**) ceftazidime (CTZ), with alpha values of 0.05 and a threshold value of 2.0

LEfSe was used to identify the features at the genus level that were differentially abundant between the two groups of patients to assess the gut bacterial signatures specific to each group of treated patients and control group (Figs. 5 and S3). *Ruminococcus* was the most abundant bacterial marker in the gut microbiota of patients treated with AMC continuous compared to the gut microbiota of patients treated with AMC bolus while *Sellimonas* was the most abundant bacterial marker (Fig. 5A). Only three bacterial markers (*Escherichia-Shigella*, *Parabacteroides* and uncultered bacteria of *Eggerthellaceae*) were identified in the gut microbiota of patients treated with CTZ continuous compared to the gut microbiota of patients treated with CTZ bolus wherein five bacterial markers were distinguished (*Eubacterium*, *Tyzzerella*, *Clostridium*_sensu_stricto_1, *Negativibacillus* and *Lachnospiraceae*-UC5-1-2E) (Fig. 5B). *Dorea*, *Coprococcus*, *Roseburia* and *Monoglobus* were differentially abundant in the fecal microbiota of the control group than in the fecal microbiota of each group of treated patients. While, *Enterococcus*, *Lachnoclostridium* and *Sellimonas* were significantly higher in the fecal microbiota of treated patients than in the fecal microbiota of the control group (Fig. S3, Table S3).

#### Effects of antibiotic administration on the functional profile of gut bacterial communities of patients with iDFUs

**Fig. 6:**
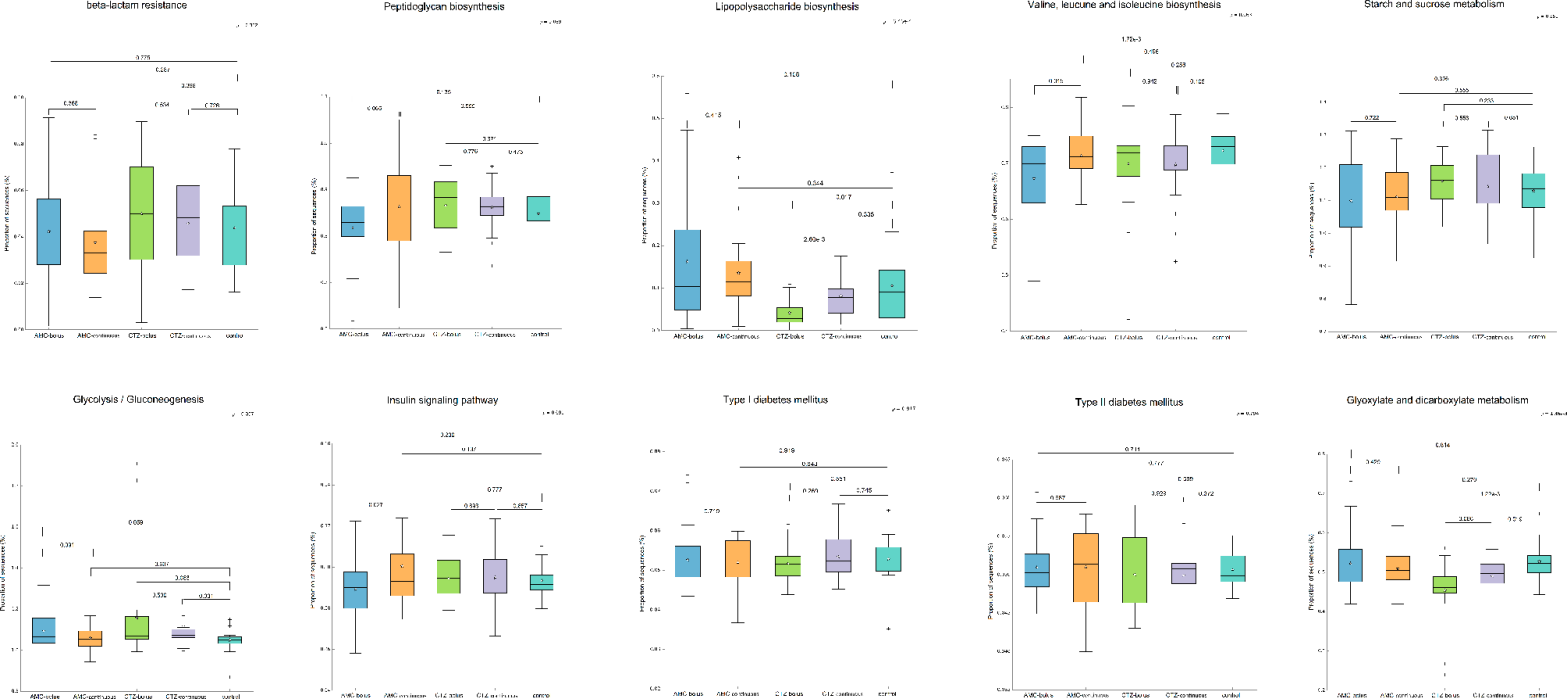
Selected KEGG functional pathways at level 3 predicted in the fecal microbiomes of control participants and patients with iDFUs treated with amoxicillin/clavulanic acid (AMC) and ceftazidime (CTZ) using bolus and continuous administration modes. p-value ≤ 0.05 was considered statistically significant

PICRUSt2 was used to determine the effect of the administration mode of antibiotics on functional pathways in the intestinal microbiota of patients with iDFUs. Results showed significant differences in gut microbiomes of patients treated using bolus and continuous administration of both antibiotics - AMC and CTZ (Figs. 6 and S4-S9, Table S4). KEGG functional pathway analysis predicted many microbial functional genes related to “metabolism”, “environment information processing”, “genetic information processing”, “cellular processes”, “organismal systems” and “human diseases” in the intestinal microbiota patients.

In patients treated with AMC, 24 pathways were significantly enriched using bolus mode and 26 pathways were significantly enhanced using continuous mode (Fig. S4). Functional pathways were more disturbed in the gut microbiome of patients treated using bolus administration form (52 pathways) than in patients treated using continuous administration form (21 pathways) compared to the control group (Figs. S5 and S6). In patients treated with CTZ, 2 pathways were significantly higher using bolus mode and 24 pathways were significantly elevated using continuous mode (Fig. S7). Compared to control groups, number of dysregulated pathways was higher in the gut microbiome of patients treated using bolus administration form (36 pathways) than in patients treated using continuous administration form (27 pathways) (Figs. S8 and S9).

Some KEGG pathways related to antibiotic resistance (“beta-lactam resistance”), diabetes (“type I diabetes mellitus”, “type II diabetes mellitus”, “insulin signaling pathways”), carbohydrate metabolism (“glycolysis/glucogenesis”, “glyoxylate and dicarboxylate metabolism”, “starch and sucrose metabolism”), glycan biosynthesis and metabolism (“lipopolysaccharide biosynthesis”, “peptidoglycan biosynthesis”), and amino acid metabolism (“valine leucine and isoleucine biosynthesis”) were particularly analyzed (*P* < 0.05; Fig. 6).

## Discussion

Globally, DFIs are the most predominant cause of hospitalization and lower extremity amputations [1–4] accompanied by high patient morbidity and mortality and cost burdens [7]. Antimicrobial therapies are usually prescribed in order to treat DFIs and to prevent DF complications [9, 10]. The commonly used form of administering antibiotics is obviously an intermittent bolus regime. Infrequently, continuous intravenous infusion administration is also used form. The use of antibiotics alters the bacterial diversity, taxonomic composition and functional capacity of the human gut microbiota, inducing gut dysbiosis [17, 19, 55–61]. Several studies have shown that gut microbiota dysbiosis has been linked to the development of DM [27–30, 32, 62]. Moreover, gut microbiota may have a potential role in diabetic wound healing through the gut microbiota-skin axis [46]. However, the link between DFIs pathogenesis, the mode of antibiotic treatment, and its impact on the functionality and diversity of the gut microbiome is not well established. In this study, we aimed to investigate how antibiotic treatment, especially the form of antibiotic administration (bolus vs. continuous), influences the gut microbiota composition and functionality. To our knowledge, this is the first study to assess the effect of antibiotic administration mode on the gut microbiome of patients with iDFUs based on metagenomic sequencing methods.

In this study, we have analyzed the gut microbiome of hospitalized patients with iDFUs, who were treated with amoxicillin/clavulanic acid or ceftazidime via bolus or continuous administration regimes. As with the majority of studies, the design of the current study is subject to limitations. From 60 patients with iDFUs, only 35 patients provided stool samples at all three times of collection. That decreases the study lot but does not affect the sufficiency of sample size for statistical measurements for microbiome analysis.

Alpha diversity of gut microbiome of patients was not affected by antibiotic treatments using both administration modes (Fig. 2). Some significant differences in diversity evenness (pielou evenness and simpson indices) but not in diversity richness (shannon and chao1 indices) were observed between the control group consisting of patients with T2DM without iDFUs and antibiotic pretreatment and study patients with iDFUs. Significant differences in beta diversity were noticed between the control group and two administration forms: bolus and continuous, in both study groups treated by AMC and CTZ (Fig. 3). However, beta diversity was not significantly affected at different times of samples´ collection (V0, V2 and V5) in both antibiotics treatments using both administration forms (Fig. S1). PCoA analysis showed that samples from the same patient remained more similar to each other than to those from other patients which could be related to the individual-specific response of human gut microbiota to antibiotics based on baseline microbiota composition observed in comparable studies [63, 64].

*Lactobacillus* was significantly higher in fecal microbiome of patients treated with AMC using bolus administration form while the abundance of *Bifidobacterium* was higher in fecal microbiome of all patients treated with CTZ (Fig. 4). Increased evidence indicates that consuming probiotics, including *Lactobacillus* and *Bifidobacterium*, is associated with wound healing in DFIs in humans [45], and rats [65, 66]. However, probiotics were not prescribed routinely to our study subjects. Additionally, it was shown that the use of metformin increased the relative abundance of *Bifidobacterium* and *Lactobacillus* [67]. Findings showed that *Lactobacillus* was enriched in the gut microbiome of diabetic patients from different global populations [68–71]. Abundance of *Bifidobacterium* was associated with ketoacidosis in T2DM [72]. *Enterococcus* was significantly higher in fecal microbiome of patients treated with CTZ compared to the fecal microbiome of patients treated with AMC and it was reduced intensely after 10 weeks of CTZ treatment using both administration forms (Fig. 4). *Enterococcus* was associated with patients with T2DM [73–75] and T1DM in rats [76]. The abundance of *Lactobacillus*, *Bifidobacterium* and *Enterococcus* varied greatly between investigations that studied antibiotic-induced changes in the human gut microbiota, ranging from no change to significant changes [75].

The functional profiling results showed that the gut microbiota of patients were involved more in metabolic pathways, which is consistent with the metabolic disease of diabetes. We highlighted statistically significant (*P* < 0.05) differentially abundant pathways of intestinal bacterial genes that discriminate between groups of patients with iDFUs, including “type I diabetes mellitus” and “type II diabetes mellitus” pathways, “insulin signaling pathway”, “beta-lactam resistance”, “glycolysis/glucogenesis pathway”, “glyoxylate and dicarboxylate metabolism”, “starch and sucrose metabolism”, “valine, leucine and isoleucine biosynthesis”, and “peptidoglycan biosynthesis”, “lipopolysaccharide biosynthesis”. Similarly, Wang et al. have reported that “starch and sucrose metabolism”, “insulin signaling pathway” and “peptidoglycan biosynthesis” were mainly enriched in the gut microbiota of patients with DFIs [77]. We have observed a significant difference in the predicted pathway “insulin signaling pathway” between bolus and continuous administration modes in patients treated with AMC with p values of *P* = 0.027. It could explain not only the impact on DM induction but also on wound healing in DFUs via the modulatory role of insulin [78–81]. Wang et al. suggested that intestinal *Streptococcus* might be involved in the pathogenesis of DFIs by regulating the insulin pathway [77]. That is consistent with our results, the relative abundance of *Streptococcus* was higher in the gut microbiota of patients treated with AMC continuously while the genes related to the insulin signaling pathway were enriched in their gut microbiome (Figs. 4 and 6). “Glycolysis/glucogenesis” pathway in the fecal microbiota of patients treated with continuous CTZ mode was significantly higher than in the control group (*P* = 0.031). A study on Japanese diabetic patients showed that the upregulation of the insulin signaling pathway and glycolysis/gluconeogenesis was correlated with HbA1c and fasting plasma glucose levels [82]. Antibiotic therapy enhanced insulin signaling in diabetes-prone C57BL/6J mice fed a high-fat diet [83]. “Glyoxylate and dicarboxylate metabolism” pathway in the gut microbiome of the control diabetic patients was significantly higher than in patients treated with CTZ using both administration modes (*P* = 1.22e-3, *P* = 0.019) (Fig. 6). Similarly, an augmented abundance of reactions in glyoxylate and dicarboxylate metabolism was detected in the cohorts of the gut microbiome of humans with T2DM [84]. Elevated levels of glyoxylate were linked with hyperglycemia diabetes-associated complications and it can thus be considered as an early marker in diabetes diagnosis [85]. Glyoxylate and dicarboxylate metabolism increased in obese Swedish subjects with metabolic disorders including obesity and T2DM [84]. This metabolic pathway was upregulated under antibiotic stress and enhanced the pathogenesis and virulence of bacteria like *Escherichia coli* and *Pseudomonas aeruginosa* [86, 87]. The proportion of *Escherichia-Shigella* was significantly decreased in patients treated with CTZ therefore “Glyoxylate and dicarboxylate metabolism” pathway was significantly lower in their gut microbiome (Figs. 4, 5 and 6, Table S2 and S3). The abundance of genes related to the “lipopolysaccharide biosynthesis” pathway was significantly lower in the gut microbiome of patients treated with CTZ via bolus than in the gut microbiome of patients treated with CTZ using continuous administration mode (*P* = 2.60e-3), and control participants (*P* = 0.017). Lipopolysaccharide biosynthesis was abundant in the gut microbiome of European women patients with T2DM [88], in children with T1DM [89] and in rodent models [90]. Moreover, the proportions of lipopolysaccharide biosynthesis pathway were related to inflammation and tissue damage in patients with DFIs and were higher in groups with severe DFIs than in groups with mild forms [91]. Additionally, it was demonstrated that the lipopolysaccharide-producing bacteria were significantly enhanced in patients with T2DM [88]. Members of *Escherichia-Shigella* could produce lipopolysaccharide which participates in the inflammation of diabetics [73, 76]. Similarly, our results show a positive correlation between the relative abundance of *Escherichia-Shigella* and the proportion of genes related to lipopolysaccharide biosynthesis. The proportion of *Escherichia-Shigella* and genes related to lipopolysaccharide biosynthesis were significantly lower in the gut microbiota of patients treated with CTZ using bolus administration mode compared with the gut microbiota of patients treated with continuous CTZ and control group (Figs. 4, 5 and 6, Table S2 and S3). “Valine, leucine and isoleucine biosynthesis” pathway was significantly lower in the gut microbiome of patients treated with AMC using bolus administration mode compared to patients treated with AMC using continuous mode (*P* = 0.015) and control participants (*P* = 1.72e-3). Findings showed that the elevated levels of BCAAs (Branched-chain amino acids), or valine, isoleucine and leucine, are associated with obesity and diabetes, and contribute to insulin resistance [92, 93].

“Beta-lactam resistance” was detected in the gut microbiome of all studied patients however no significant difference was observed using both antibiotics via applying two administration forms (Fig. 6). Generally, antibiotic resistance genes were identified in patients with DFIs which mainly included beta-lactam resistance genes [94]. Extended-spectrum beta-lactamase (ESBL)-producing *Enterobacteriaceae* were detected in the gut microbiota of patients admitted to European hospitals [95, 96]. ESBL-producing *Enterobacteriaceae* are a leading cause of antibiotic resistance and treatment failure in Europe [97]. Translocation of bacteria and bacterial products (like beta-lactamase) from the gut to the bloodstream may occur in case of dysfunction of intestinal barrier permeability or “leaky gut” [98, 99] leading to suggest the potential transfer of ESBL-producing *Enterobacteriaceae* or its products to the wound via gut-skin axis and causing failure of wound healing in patients with iDFUs [46]. Furthermore, it was reported that long exposure to antibiotics was associated with an increased risk of T2DM [19, 21, 100, 101]. Early childhood antibiotic treatment was associated with an increased risk of T1DM [102, 103].

“Cellular processes” and “environmental information processing” were also perturbed in all patients due to the use of antibiotics. Alterations in pathways like “membrane transport”, “membrane and intracellular structural molecules”, “pores ion channels signal transduction” and “signaling molecules and interaction” lead to an increase in intestinal barrier permeability, often referred to as “the leaky gut”. Genes related to membrane transport were mostly upregulated in the gut microbiome of patients treated with antibiotics using bolus administration form (Figures S4, S5, S7 and S8, Table S4). Various investigations have revealed that antibiotic administration caused gut microbial dysbiosis, impaired intestinal morphological development, and disrupted intestinal barrier function [104–106]. Moreover, intestinal barrier dysfunction caused by reduced intestinal integrity enhances the translocation of microbial components and immune stimulants into the bloodstream which might promote systemic inflammation leading to the progression of diabetes complications [107–111]. Several studies indicated the association between intestine hyper-permeability and the progression and development of diabetes [110–113]. These findings are comparable to reported suggestions about the linkage between gut dysbiosis and its relation with the pathogenesis of DFIs and delayed wound healing [43]. Restoring gut microbiome balance and diversity via probiotics could be a promising strategy in the prevention of the negative impact of antibiotics on gut microbiota diversity and its functionality and indirectly affecting wound healing in patients with DFIs [42, 43, 46].

## Conclusion

This study shows the differential effects of antibiotic administration mode on the gut microbiome composition and functionality. Both bolus and continuous administration modes affect the gut microbiome of treated patients with two kinds of beta-lactams without any particular preference between the two administration forms. This study revealed the bacterial markers and potential metabolic signatures associated with each administration mode in patients with iDFUs. Hyperpermiability of the intestinal barrier was correlated with bolus administration form. Genes related to “insulin signaling pathways”, “lipopolysaccharide biosynthesis”, and “valine leucine and isoleucine biosynthesis” were associated with continuous administration form. By building on these key findings, subsequent studies using large size of subject groups can drive significant advancements in the field of antibiotic therapy, ultimately leading to improved health outcomes and enhanced quality of life for patients. Provided results will help healthcare professionals choose which administration form of antibiotics is suitable for patients with infected diabetic foot ulcers to minimize the adverse events in order to prevent the number of lower amputations and reduce the patient and healthcare burden of diabetes-related foot disease.

## Electronic supplementary material

Below is the link to the electronic supplementary material.

Supplementary Material 1: Principal Coordinate Analysis (PCoA) plots based Bray Curtis distance of fecal microbiome of patients with iDFUs treated with: A) amoxicillin/clavulanic acid (AMC) using bolus administration mode, B) amoxicillin/clavulanic acid (AMC) using continuous administration mode, C) ceftazidime (CTZ) using bolus administration mode, D) ceftazidime (CTZ) using continuous administration mode. Label numbers correspond to the patient ID. Dissimilarity analysis between the two groups was performed using Adonis with permutation 999. The confidence ellipses were traced in the 95% confidence. p-value ≤ 0.05 was considered statistically significant.

Supplementary Material 2: Relative abundance of bacterial populations at phylum level of fecal microbiomes of A) control participants and patients with iDFUs treated with amoxicillin/clavulanic acid (AMC) and ceftazidime (CTZ) using bolus and continuous administration modes at different times of collection V0 (before hospitalization), V2 (one week after hospital admission) and V5 (two months after hospital discharge).

Supplementary Material 3: Linear discriminant analysis effect size (LEfSe) of taxa at genus level in fecal microbiomes from control group compared to A) the fecal microbiome of patients with iDFUs treated with amoxicillin/clavulanic acid (AMC) using bolus administration mode, B) the fecal microbiome of patients with iDFUs treated with amoxicillin/clavulanic acid (AMC) using continuous administration mode C) the fecal microbiome of patients with iDFUs treated with ceftazidime (CTZ) using bolus administration mode and D) the fecal microbiome of patients with iDFUs treated with ceftazidime (CTZ) using bolus administration mode, with alpha values of 0.05 and a threshold value of 2.0.

Supplementary Material 4: Predicted functional KEGG pathways at level 3 of the fecal microbiomes of patients with iDFUs treated with amoxicillin/clavulanic acid (AMC) using bolus and continuous administration modes. p value ≤ 0.05 was considered statistically significant.

Supplementary Material 5: Predicted functional KEGG pathways at level 3 of the fecal microbiomes of control participants and patients and with iDFUs treated with amoxicillin/clavulanic acid (AMC) using bolus administration modes. p value ≤ 0.05 was considered statistically significant.

Supplementary Material 6: Predicted functional KEGG pathways at level 3 of the fecal microbiomes of control participants and patients and with iDFUs treated with amoxicillin/clavulanic acid (AMC) using continuous administration modes. p value ≤ 0.05 was considered statistically significant.

Supplementary Material 7: Predicted functional KEGG pathways at level 3 of the fecal microbiomes of patients with iDFUs treated with ceftazidime (CTZ) using bolus and continuous administration modes. p value ≤ 0.05 was considered statistically significant.

Supplementary Material 8: Predicted functional KEGG pathways at level 3 of the fecal microbiomes of control participants and patients and with iDFUs treated with ceftazidime (CTZ) using bolus administration modes. p value ≤ 0.05 was considered statistically significant.

Supplementary Material 9: Predicted functional KEGG pathways at level 3 of the fecal microbiomes of control participants and patients and with iDFUs treated with ceftazidime (CTZ) using continuous administration modes. p value ≤ 0.05 was considered statistically significant

Supplementary Material 10: List of control participants (*n* = 17) and patients and with iDFUs (*n* = 49) treated with amoxicillin/clavulanic acid (AMC) and ceftazidime (CTZ) using bolus and continuous administration modes.

Supplementary Material 11: The relative abundances of the bacterial community based on the rarefied table at phylum, family and genus levels in the fecal microbiome of control participants and patients with iDFUs treated with amoxicillin/clavulanic acid (AMC) and ceftazidime (CTZ) using bolus and continuous administration modes at different times of collection V0 (before hospitalization), V2 (one week after hospital admission) and V5 (two months after hospital discharge).

Supplementary Material 12: List of significantly abundant bacteria at genus levels in the fecal microbiome of control participants and patients with iDFUs treated with amoxicillin/clavulanic acid (AMC) and ceftazidime (CTZ) using bolus and continuous administration modes.

Supplementary Material 13: Predicted functional KEGG pathways at level 3 in the fecal microbiomes of control participants and patients with iDFUs treated with amoxicillin/clavulanic acid (AMC) and ceftazidime (CTZ) using bolus and continuous administration modes. p-value ≤ 0.05 was considered statistically significant.

## Data Availability

The nucleotide sequences have been submitted in the NCBI database in Sequence Read Archive (SRA) SUB14188629 under BioProject ID: PRJNA1071358.

## Abbreviations

ABC transporters: ATP-Binding Cassette transporters
AMC: Amoxicillin/clavulanic acid
BCAAs: Branched-Chain Amino Acids
CI: Confidence Interval
CTZ: Ceftazidime
DFIATIM trial: Diabetic Foot Infection treated with ATBs and its Impact on gut Microbiota
DFIs: Diabetic Foot Infections
DFUs: Diabetic Foot Ulcers
DM: Diabetes Mellitus
ESBL-producing Enterobacteriaceae: Extended-spectrum beta-lactamase-producing Enterobacteriaceae
FMT: Fecal Microbiota Transplantation
HbA1c: Hemoglobin A1C (glycated hemoglobin)
iDFUs: Infected Diabetic Foot Ulcers
IWFDF/IDSA: International Working Group on the Diabetic Foot/ Infectious Diseases Society of America
KEGG: Kyoto Encyclopedia of Genes and Genomes
LefSe: Linear discriminant analysis with effect Size
MIC: Minimum Inhibitory Concentration
PCoA: Principal Coordinate Analysis
PICRUSt2: Phylogenetic Investigation of Communities by Reconstruction of Unobserved States version2
PK/PD: Pharmacokinetic/Pharmacodynamics
PTS: Phosphotranspherase
QIIME2: Quantitative Insights Into Microbial Ecology version 2
T1DM: Type 1 Diabetes Mellitus
T2DM: Type 2 Diabetes Mellitus
V0: Hospital day 0_ inclusion visit-V0
V2: Hospital day 7_visit -V2
V5: 8 weeks_end of study-V5
