# Supplementary figures and images for: The effect of the administration form of antibiotic therapy on the gut microbiome in patients with infected diabetic foot ulcers - DFIATIM trial
Source: BMC Microbiol. 2025 May 28;25:339. doi: 10.1186/s12866-025-04041-0 (PMC12117690; doi:10.1186/s12866-025-04041-0)

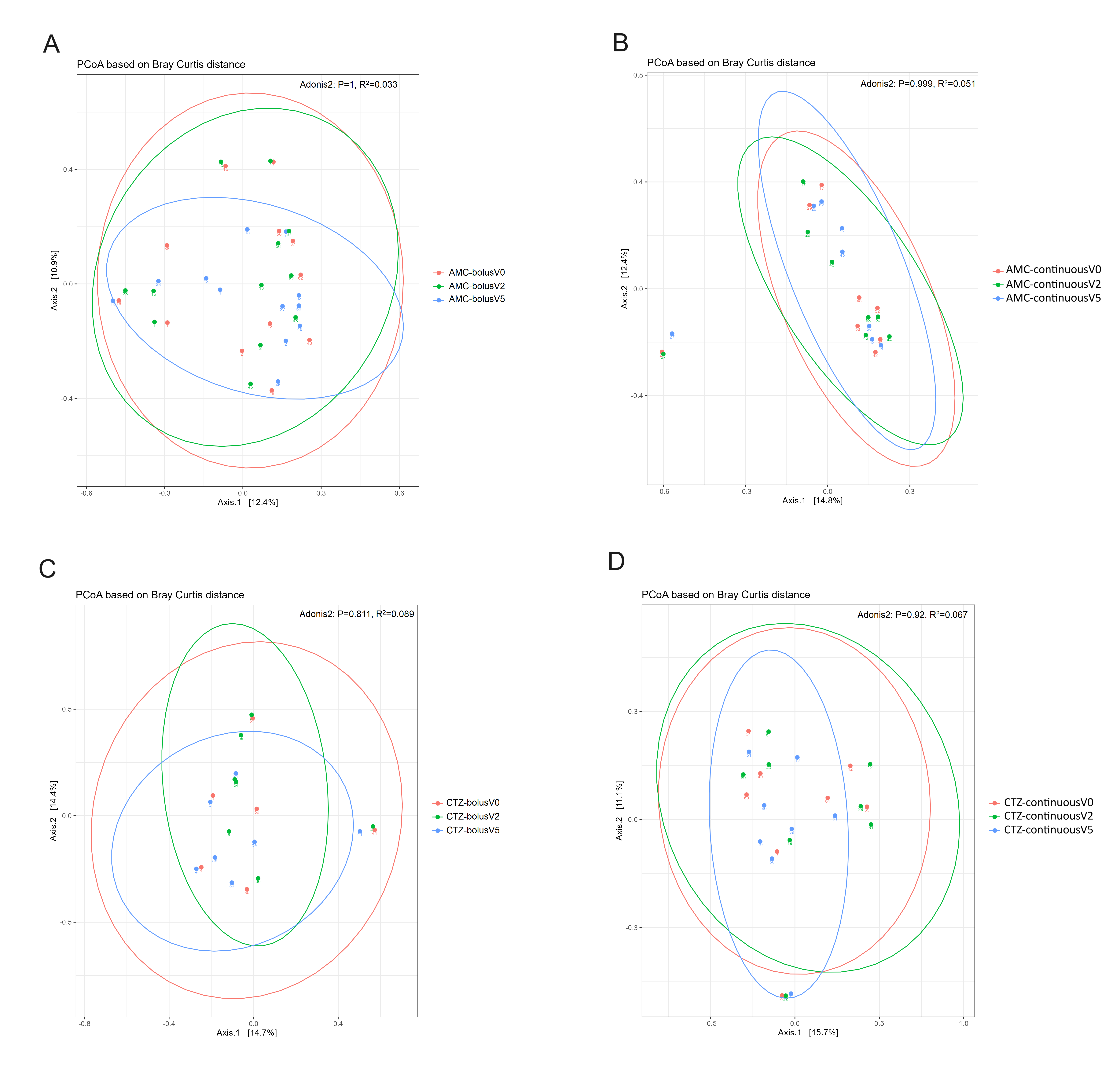

Supplement: Supplementary file 1 — Supplementary Material 1: Principal Coordinate Analysis (PCoA) plots based Bray Curtis distance of fecal microbiome of patients with iDFUs treated with: A) amoxicillin/clavulanic acid (AMC) using bolus administration mode, B) amoxicillin/clavulanic acid (AMC) using continuous administration mode, C) ceftazidime (CTZ) using bolus administration mode, D) ceftazidime (CTZ) using continuous administration mode. Label numbers correspond to the patient ID. Dissimilarity analysis between the two groups was performed using Adonis with permutation 999. The confidence ellipses were traced in the 95% confidence. p-value ≤ 0.05 was considered statistically significant. [file 12866_2025_4041_MOESM1_ESM.png]

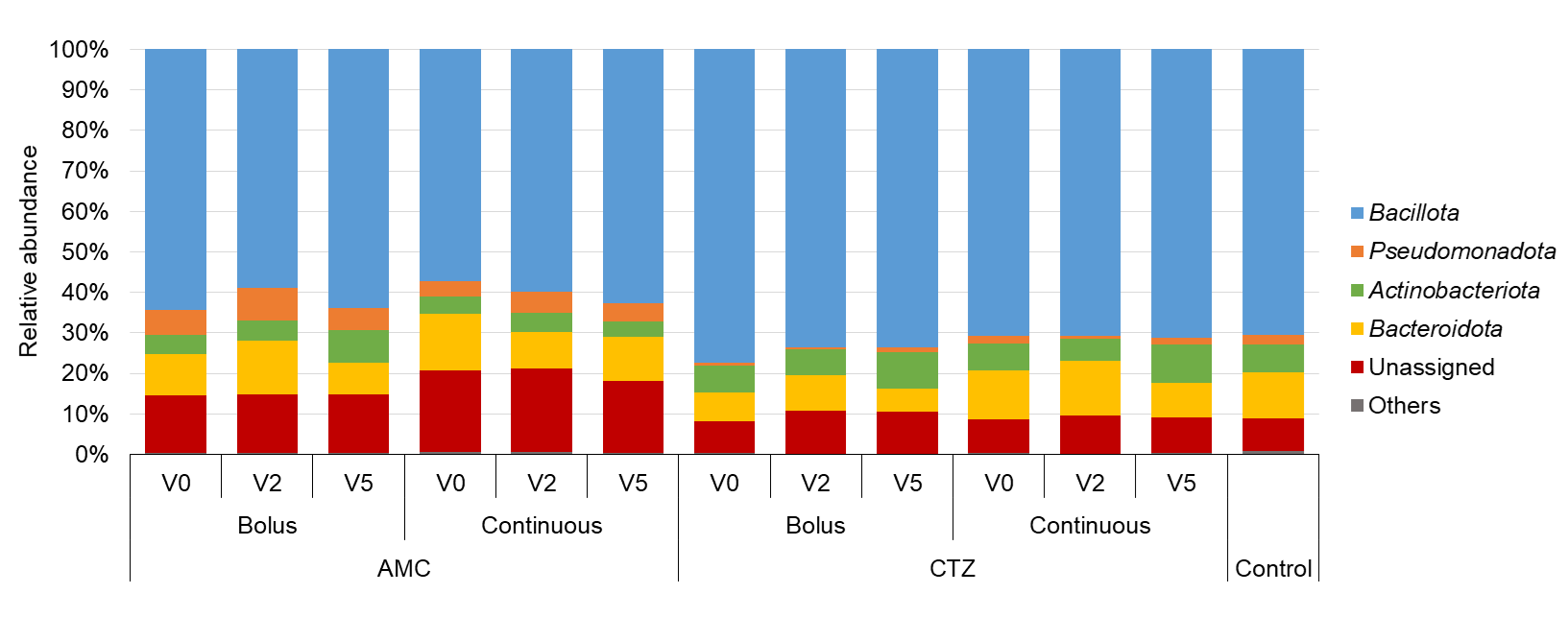

Supplement: Supplementary file 2 — Supplementary Material 2: Relative abundance of bacterial populations at phylum level of fecal microbiomes of A) control participants and patients with iDFUs treated with amoxicillin/clavulanic acid (AMC) and ceftazidime (CTZ) using bolus and continuous administration modes at different times of collection V0 (before hospitalization), V2 (one week after hospital admission) and V5 (two months after hospital discharge). [file 12866_2025_4041_MOESM2_ESM.png]

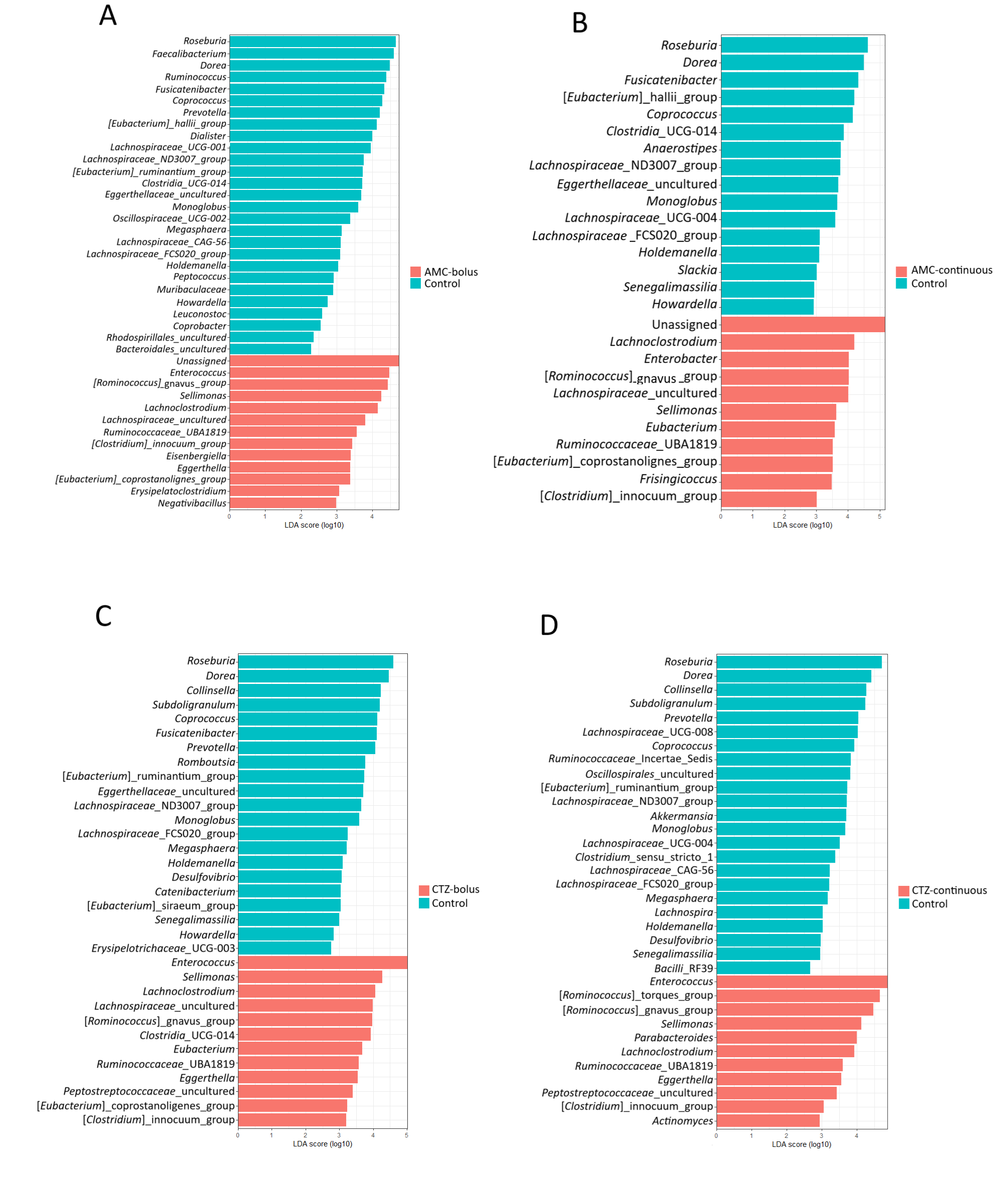

Supplement: Supplementary file 3 — Supplementary Material 3: Linear discriminant analysis effect size (LEfSe) of taxa at genus level in fecal microbiomes from control group compared to A) the fecal microbiome of patients with iDFUs treated with amoxicillin/clavulanic acid (AMC) using bolus administration mode, B) the fecal microbiome of patients with iDFUs treated with amoxicillin/clavulanic acid (AMC) using continuous administration mode C) the fecal microbiome of patients with iDFUs treated with ceftazidime (CTZ) using bolus administration mode and D) the fecal microbiome of patients with iDFUs treated with ceftazidime (CTZ) using bolus administration mode, with alpha values of 0.05 and a threshold value of 2.0. [file 12866_2025_4041_MOESM3_ESM.png]
